# Incidence and Outcomes of 72-Hour Return Visits in a Paediatric Emergency Department: A One-Year Retrospective Observational Study

**DOI:** 10.7759/cureus.114765

**Published:** 2026-08-18

**Authors:** Abdulmohsen L Alsolami, Omar A Alawni, Yasir M ALbahli, Yara S AlHabib, Elsharif A Bazie

**Affiliations:** 1 Emergency Department, Security Forces Hospital, Riyadh, SAU; 2 Emergency Department, University of El Imam El Mahdi, Kosti, SDN

**Keywords:** emergency medicine, pediatric medicine, pediatrics emergency, return visit, unscheduled revisit

## Abstract

Background

Emergency department (ED) return visits within 72 hours are a healthcare quality indicator and a measure of system efficiency. Paediatric Emergency Department (PED) revisits reflect both disease progression and healthcare utilisation patterns. Understanding revisit characteristics and outcomes can guide targeted quality improvement strategies.

Objectives

The primary objective of this study was to describe the pattern of PED return visits within 72 hours.

Methods

This study was conducted at the PED of Security Forces Hospital in Riyadh, Saudi Arabia, between January and December 2024. This was a retrospective descriptive study. The study population included paediatric patients (<14 years old) who revisited the PED within 72 hours of the initial (index) visit, and data were collected in an Excel sheet from the PED database system. In Saudi Arabia, pediatric age is 14 years old and below.

Data variables included demographics, Canadian Triage and Acuity Scale (CTAS) category, diagnosis (ICD-10), visit timing, and outcomes.

Results

The total number of PED visits during the study period was 69,288, of which 5081 (7.33%) were revisits. Of the total revisits (5081), 76.72% were within 72 hours. CTAS Category 3 accounted for the largest proportion of revisits, followed by CTAS Category 2. Most revisits occurred during evening shifts, with seasonal peaks in October, December, and January. The most common revisit diagnoses were respiratory tract and gastrointestinal complaints.

Conclusion

Most revisits involved young children and were due to respiratory infections. Health education for the high-risk group and improved initial triage may reduce revisit rates to PED.

## Introduction

The emergency department (ED) is typically a high-setting area with a constant flow of new and revisiting patients and no follow-up after discharge by the physician. In this busy place, high-risk patients may be at risk of serious outcomes [1,2].

Unscheduled return visits (URVs) or return visits are defined as visits to the ED within 72 hours of the first visit to the ED [3].

Return visits give less time for the physician to evaluate each patient and make the doctors pay much more attention to the returning patients because there is a risk of progression of the previous illness or fear of missing or wrong diagnoses. This is why returning patients are more frequently hospitalised and have upgraded triage in the triage area. Moreover, blood work is performed more frequently for them than for patients on their first visit [4]. On the other hand, overcrowding in the ED is the main driver of increased ER return visits. Nevertheless, reducing overcrowding did not decrease RV rates in many hospitals [5].

EDs worldwide are overflowing with patients, and patients suffer from prolonged lengths of stay and waiting times [6]. Like other countries, the pediatrics EDs in Saudi Arabia serve as a crucial point of access to medical care [6]. Despite ongoing efforts by the Ministry of Health (MOH) and other governmental sectors to decrease the load on EDs by introducing urgent care facilities in primary healthcare centres in most areas, strict laws and cultural changes are needed to achieve such goals [7].

This study aimed to characterise the patterns of URVs to Paediatric Emergency Departments (PEDs) by examining the timing of these visits, patient age distribution, Canadian Triage and Acuity Scale (CTAS) levels, and diagnoses associated with the second visit. By clarifying these patterns, this study aims to help healthcare providers develop guidelines for improving services.

## Materials and methods

We conducted a retrospective, descriptive, observational study at the PED of the Security Forces Hospital in Riyadh, Saudi Arabia. The PED is open 24 hours a day and staffed by certified paediatric emergency consultants, physicians, residents, and nursing teams.

The study is for a one-year duration, conducted from January 1 to December 31, 2024. Inclusion criteria for the study are all paediatric patients younger than 14 years who returned to the PED within 72 hours of discharge from their index visit. Patients were excluded if they were 14 years or older, revisited the PED outside the study period (before January 1 or after December 31, 2024), attended follow-up with their pediatrician/primary care provider appointments or lab results, returned to the PED after 72 hours of their index visit, triaged CTAS 5, or had incomplete or missing data in the hospital records system.

Authorized personnel extracted the data from the hospital’s Track Care system for the study based on the inclusion and exclusion criteria.

The primary outcome assessed was the return visit rate to the PED within 72 hours, while secondary outcomes included the CTAS level, diagnoses at revisit, and timing of return.

Triage categories were based on the Canadian Triage and Acuity Scale (CTAS; 1-5) [8], and diagnoses were classified using ICD-10 codes (International Classification of Diseases, 10th Revision) [9].

The age-group distributions and CTAS-level change analyses were done using the chi-square test to compare categorical variables. For age-group distributions, a chi-square statistic of χ2= 343.89 (p < 0.001) was observed. For CTAS-level changes, a chi-square statistic of X² = 77.29 (p< 0.001) was observed. A p-value of <0.05 was considered statistically significant.

This study was conducted in accordance with the guidelines and regulations of the research committee at the Security Forces Hospital Program, Riyadh, Saudi Arabia (Research Number: 25-864-84). The Institutional Review Board (IRB) overseeing this research is registered with the King Abdulaziz City for Science and Technology (KACST), Kingdom of Saudi Arabia (IRB Registration Number: H-01-R-069). The data utilised in this study were obtained electronically from the hospital system; consent to participate is unnecessary according to the Institutional Review Board (IRB) regulations.

## Results

The study was a retrospective, hospital-based study conducted over one year, from January to December 2024, in which a total of 69,288 children were seen in the PED, of whom 5,081 (7.33%) returned to the PED. Of the total revisits, 76.72% occurred within 72 hours of the index visit.

Age distribution and return visits

Regarding the age groups among return visits to the PED, children under one year of age were disproportionate. Infants represent 16% of all PED visits and 23% of revisit visits to the PED within 72 hours (χ² = 48.32, p < 0.001), indicating a significantly higher return visit rate in infants. The 1-2-year-old age group's proportion of return visits was also slightly higher than their overall representation (26% among returns vs 23% overall). The rate of return to PED for the 3-6-year-old group, compared with the total return visitor population, was 24%, while this age group accounted for 32% of all PED visits overall. Children aged 12-14 years were the least likely age group to return to PED (3% among return visits vs 5% overall).

**Table 1:** Age Distribution of Overall PED Population (N = 69,288) and Return Visits (N = 5,081). PED: Paediatric Emergency Department

Age Group	Overall PED Population n (%)	Return Visits within 72 Hours n (%)	p-value
Less than one year	11,086 (16%)	1,169 (23%)	< 0.001
1 to 2 years	15,936 (23%)	1,321 (26%)	
3 to 6 years	22,172 (32%)	1,219 (24%)	
7 to 11 years	16,629 (24%)	1,219 (24%)	
12 to 14 years	3,464 (5%)	152 (3%)	

Monthly distribution of return visit trends

The highest rates of return visits within 72 hours were reported in December (8.2%), January (8.2%), and June (8.3%), whereas the lowest rate was in September (5.8%). The findings showed that return visit rates varied over the year; the variation was related to seasonal pediatric illness patterns.

**Table 2:** Monthly Distribution of Return Visits (January - December 2024) (N = 5,081).

Month	Number of Return Visits	Percentage of Total PED Visits (%)
January	587	8.2
February	336	6.3
March	356	7.3
April	338	6.8
May	438	7
June	356	8.3
July	383	8.2
August	314	6.7
September	371	5.8
October	531	7.3
November	385	7.1
December	686	8.2

Interval time of return

The most frequent return visit interval was within the first 24 hours, while about 75% of return visits occurred within 48 hours. Specifically, 1,979 patients (38.9%) returned within 24 hours, 1,759 (34.6%) within 24 to 48 hours, and 1,343 (26.4%) within 48 to 72 hours.

**Table 3:** Interval Time of Return Visit Distribution (N = 5,081).

Interval Time	Number of Patients	Percentage (%)
Within 24 hours	1979	38.9
24 to 48 hours	1759	34.6
48 to 72 hours	1343	26.4

The triage CTAS level at return vs initial visit

A notable difference between initial and return visits in the CTAS level was noticed. The percentage of patients triaged as CTAS Level 1 (most urgent) increased from 1.6% at the initial visit to 4.5% at the return visit (χ² = 97.63, p < 0.001). Similarly, the cases of CTAS Level 3 increased from 15.4% to 16.2% (χ² = 66.02, p < 0.001), and the cases of CTAS Level 4 were the majority but decreased from 76% at the initial visit to 71.8% at the return visit (χ² = 11.08, p = 0.001).

**Table 4:** CTAS Levels for Revisit and Index Visit (N = 5,081). CTAS: Canadian Triage and Acuity Scale

CTAS Level	Revisit Patients n (%)	Index Visit Patients n (%)	p-value
CTAS 1	230 (4.5%)	82 (1.6%)	< 0.001
CTAS 2	321 (6.3%)	326 (6.4%)	
CTAS 3	821 (16.2%)	782 (15.4%)	
CTAS 4	3,650 (71.8%)	3,866 (76.1%)	

Further comparison showed that 3,820 patients (75.1%) were triaged at the same level on both visits, but 579 (11.4%) were assigned a higher CTAS Level and 682 (13.4%) a lower CTAS level on their return visit.

**Table 5:** Distribution of CTAS Level Changes between Index and Return Visits (N = 5,081). CTAS: Canadian Triage and Acuity Scale

CTAS Level Change	n (%)
Upgraded CTAS Level	579 (11.4%)
Downgraded CTAS Level	682 (13.4%)
Same CTAS Level	3,820 (75.1%)

Timing of return visits to PEM data

Regarding the distribution of patients’ return visit times to PEM, most patients return during the afternoon shift (15:00-23:00; 42.2%), with a significant difference across shifts. The second most frequent time is the overnight shift (23:00-7:00; 31%), and the least frequent time is the morning shift (7:00-15:00; 26.7%). This pattern reflects the disease pattern in paediatrics, and most patients with employee parents come in after work hours.

**Table 6:** Time of Emergency Visit for Revisiting Patients (N = 5,081).

Time Interval	Percentage	Number of Patients (n)
7:00 to 15:00	26.70%	1,356
15:00 to 23:00	42.20%	2,146
23:00 to 7:00	31.00%	1,579

In the professional diagnosis distribution, acute upper respiratory tract infection was the most frequent diagnosis at revisits to the PED, accounting for 23.8% of all revisits. Other diagnoses included unspecified gastroenteritis and colitis (9.4%), acute bronchitis (7.7%), nasopharyngitis or the common cold (5.5%), and asthma (5.2%).

**Table 7:** Professional Diagnosis for Patients Visiting the PED (N = 5,081). PED: Paediatric Emergency Department

Diagnosis	Percentage	Number of Patients (n)
Acute upper respiratory tract infection	23.80%	1,210
Unspecified gastroenteritis and colitis	9.40%	480
Acute bronchitis	7.70%	392
Acute nasopharyngitis (common cold)	5.50%	280
Asthma	5.20%	263

## Discussion

The study was conducted in the PED at Security Forces Hospital, Riyadh, from January 1, 2024, to December 31, 2024. The 72-hour return visit rate in one year was 7.33%; studies in Saudi Arabia and the Gulf region reported rates ranging from 4% to 8% [10-12], whereas a 7.6% rate was reported in Canada [13].

Most visits to the PED were by children aged 3-6 years (32%). Younger children are more frequently visitors to emergency departments because of their higher susceptibility to infectious diseases and accidents. According to Nijman et al. [14], children under five years old are the most common age group to revisit the emergency department.

Our data showed that children under one year old (16%) and those aged 12-14 years (5%) followed common paediatric emergency patterns. Similarly, Burokienė et al. [15] found that infants and children under three years old, as well as those aged 3-7 years, accounted for the largest proportions of 72-hour return visits (43.3% and 31.9%, respectively).

Return visits were highest in January and December and the lowest in September (5.8%). The peak emergency visits occurred between 15:00 and 23:00 (42.2%), corresponding to after-school hours.

A study conducted in the United States of America found that ED visits were more common during hot seasons [16]. In contrast, our data showed a higher number of ER visits during cold seasons, likely due to increased viral illnesses. Goldman et al. [17] found that younger children arriving late in the evening were most likely to return to the ED.

Studies have shown that school sessions and humidity affect PED visits, with rates increasing during school days and in cold weather [18].

In Saudi Arabia, the number of emergency visits and admissions was lower in Spring, Autumn, and Summer compared to Winter. In Australia and the UK, both winter and hot weather were associated with increased paediatric ER admissions and visits [19,20].

Our data showed an increase in return visits among children under one year of age, possibly reflecting the complexity and urgency of conditions in this age group. Similarly, a Canadian study found that most PED revisitors were younger than six years [21].

In our data, 76% of return-visit patients were classified as having a CTAS score of 4 during their initial visits. The triage levels remained unchanged between the initial and return visits in 75.1%. This significant change in disease acuity (p-value <0.001) is similar to findings from several studies in the region that have reported an association between initial triage levels and return visits [22,23].

In our study, respiratory infections accounted for 23.8% of the cases, and unspecified gastroenteritis and colitis accounted for 9.4%. The literature reported that infectious and respiratory diseases are more prevalent than gastrointestinal disorders [24,25].

A study conducted in Saudi Arabia demonstrated that respiratory diseases were the most common (66.6%), followed by gastrointestinal diseases (12.0%). The common diagnosis on revisit to the emergency department may vary with seasonal and weather variations [26].

Limitations of the study

The limitations of this study include its retrospective design, the inclusion of a 14-year age group (unlike most other countries), and a single-centre scope. Future multicentre studies should compare validated revisit rates across institutions.

## Conclusions

Seventy-two-hour return visits to the PED reflect the quality of services introduced to the community and the qualifications of the staff. The young age groups are the most frequent visitors and revisitors to the PED. Monitoring the revisit data is an accurate quality indicator for paediatric emergency care.
